# Supplementary material for: Honeybee Venom Synergistically Enhances the Cytotoxic Effect of CNS Drugs in HT-29 Colon and MCF-7 Breast Cancer Cell Lines
Source: Pharmaceutics. 2022 Feb 25;14(3):511. doi: 10.3390/pharmaceutics14030511 (PMC8952811; doi:10.3390/pharmaceutics14030511)
Supplement: Supplementary file 1 [file pharmaceutics-14-00511-s001.zip › pharmaceutics-1596589-supplementary.pdf]

pharmaceutics

MDPI

Supplementary Materials: Honeybee Venom Synergistically Enhances the Cytotoxic Effect of CNS Drugs in HT-29 Colon and MCF-7 Breast Cancer Cell Lines

Diana Duarte, Soraia I. Falcão, Iouraouine El Mehdi, Miguel Vilas-Boas and Nuno Vale

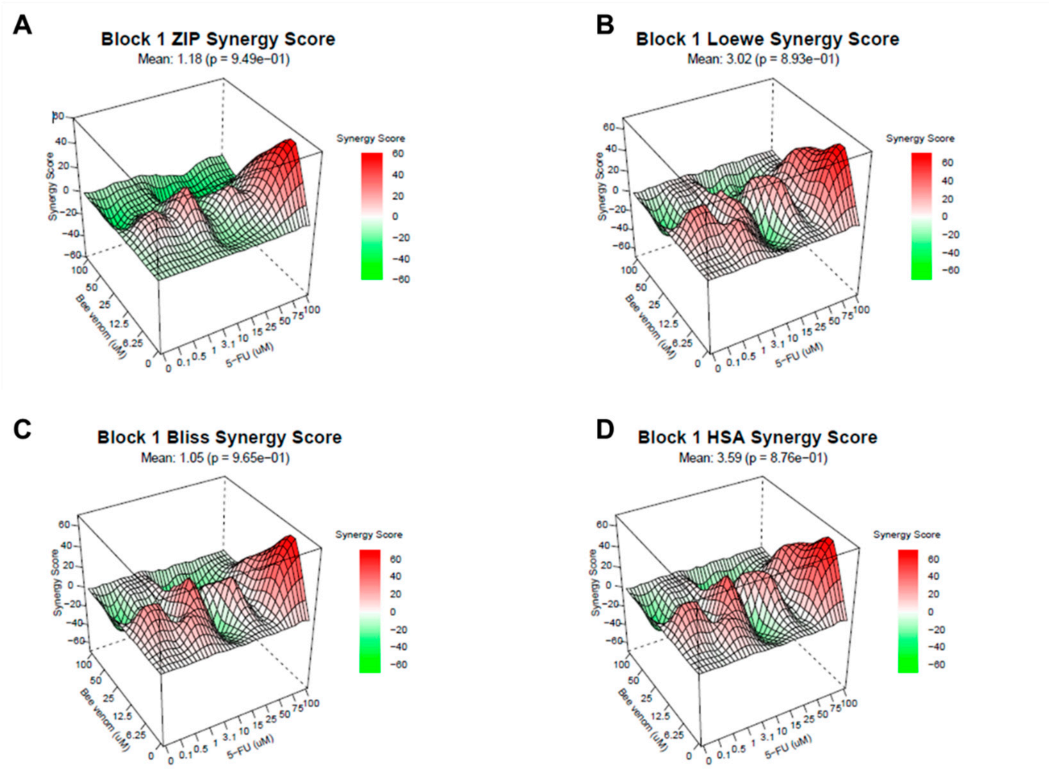

Figure S1. Synergy plots of combined treatments of 5-FU combined with honeybee venom on HT-29 colon cancer cells calculated with (**A**) ZIP; (**B**) Loewe; (**C**) Bliss and (**D**) HAS reference models. These data were obtained using SynergyFinder software. Synergy scores < −10, from −10 to 10 and > 10 indicates antagonism, additivity and synergism, respectively.

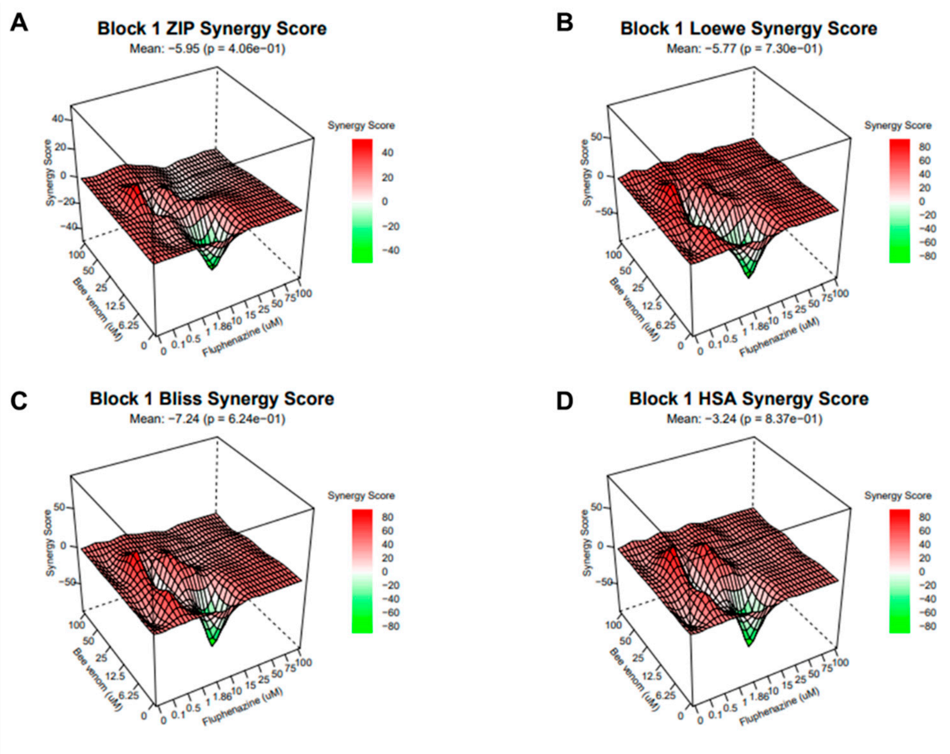

Figure S2. Synergy plots of combined treatments of fluphenazine combined with honeybee venom on HT-29 colon cancer cells calculated with (**A**) ZIP; (**B**) Loewe; (**C**) Bliss and (**D**) HAS reference models. These data were obtained using SynergyFinder software. Synergy scores < −10, from −10 to 10 and > 10 indicates antagonism, additivity and synergism, respectively.

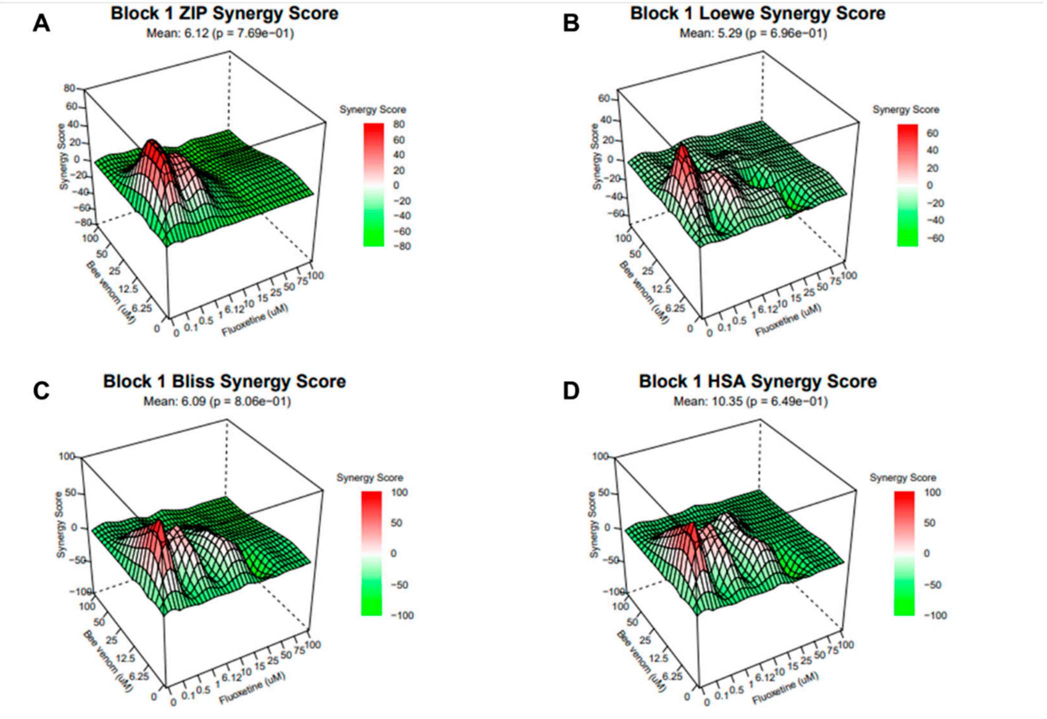

Figure S3. Synergy plots of combined treatments of fluoxetine combined with honeybee venom on HT-29 colon cancer cells calculated with (A) ZIP; (B) Loewe; (C) Bliss and (D) HAS reference models. These data were obtained using SynergyFinder software. Synergy scores < -10, from -10 to 10 and > 10 indicates antagonism, additivity and synergism, respectively.

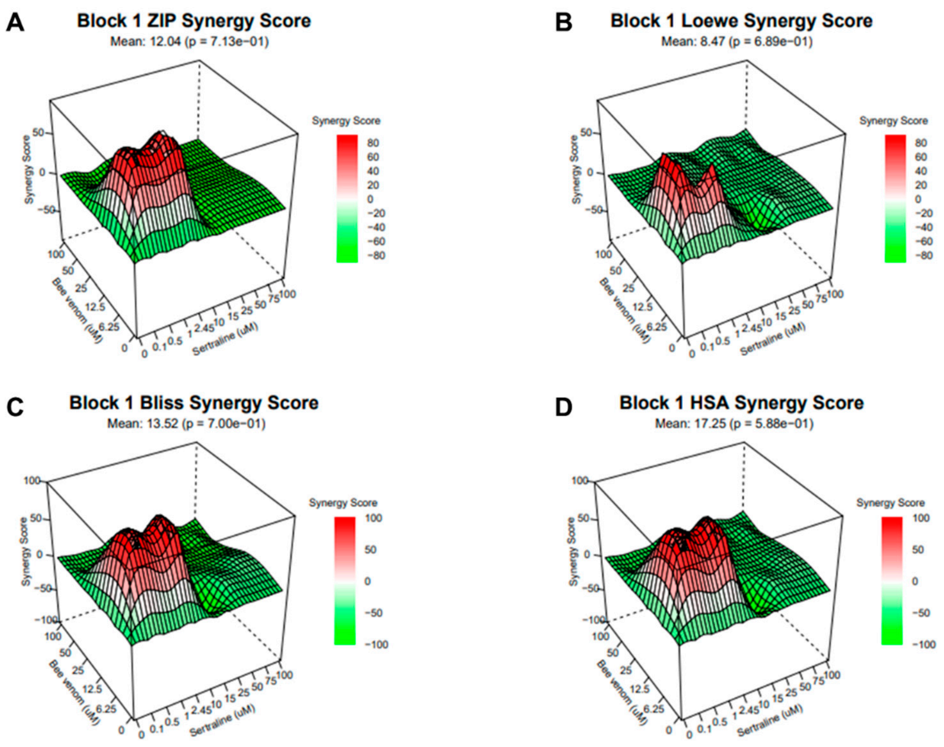

Figure S4. Synergy plots of combined treatments of sertraline combined with honeybee venom on HT-29 colon cancer cells calculated with (A) ZIP; (B) Loewe; (C) Bliss and (D) HAS reference models. These data were obtained using SynergyFinder software. Synergy scores < -10, from -10 to 10 and > 10 indicates antagonism, additivity and synergism, respectively.

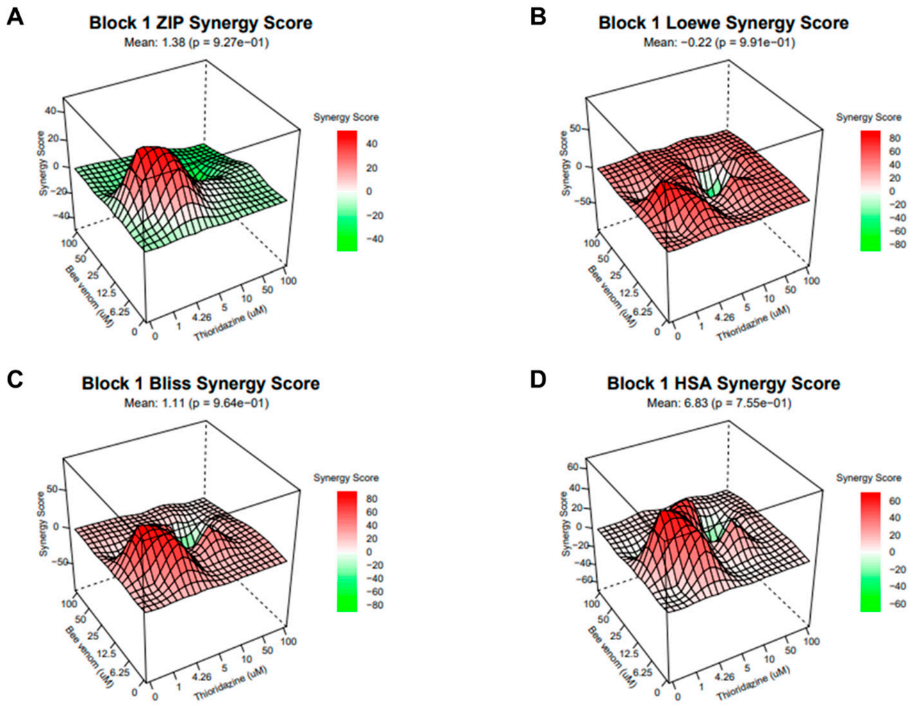

Figure S5. Synergy plots of combined treatments of thioridazine combined with honeybee venom on HT-29 colon cancer cells calculated with (A) ZIP; (B) Loewe; (C) Bliss and (D) HAS reference models. These data were obtained using SynergyFinder software. Synergy scores < -10, from -10 to 10 and > 10 indicates antagonism, additivity and synergism, respectively.

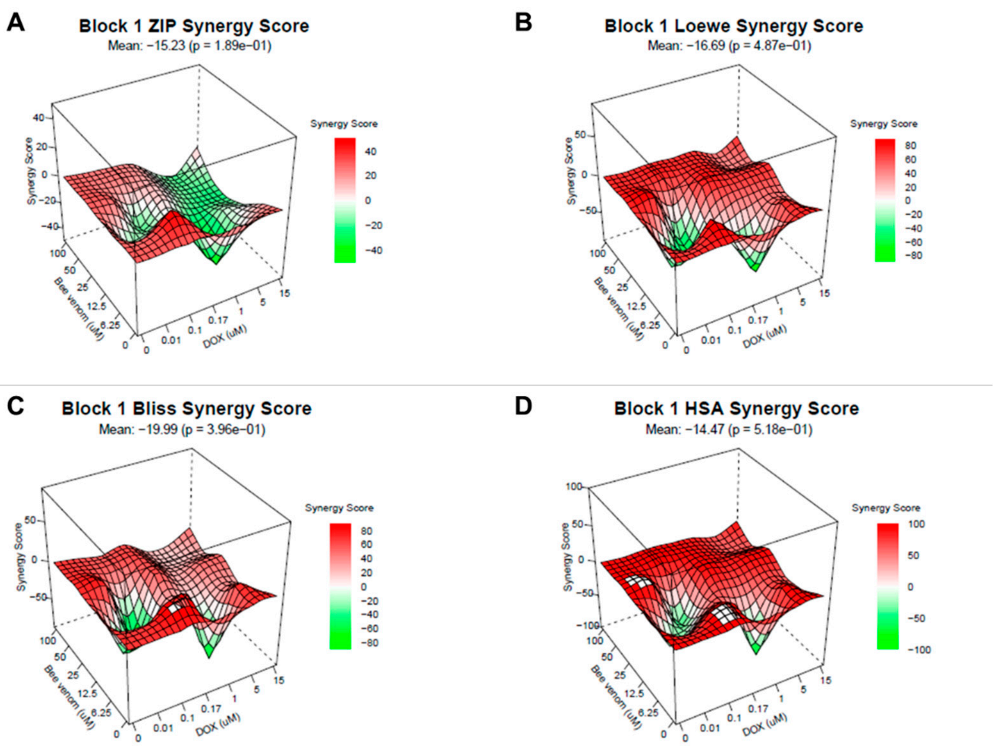

Figure S6. Synergy plots of combined treatments of DOX combined with honeybee venom on MCF-7 breast cancer cells calculated with (A) ZIP; (B) Loewe; (C) Bliss and (D) HAS reference models. These data were obtained using SynergyFinder software. Synergy scores < -10, from -10 to 10 and > 10 indicates antagonism, additivity and synergism, respectively.

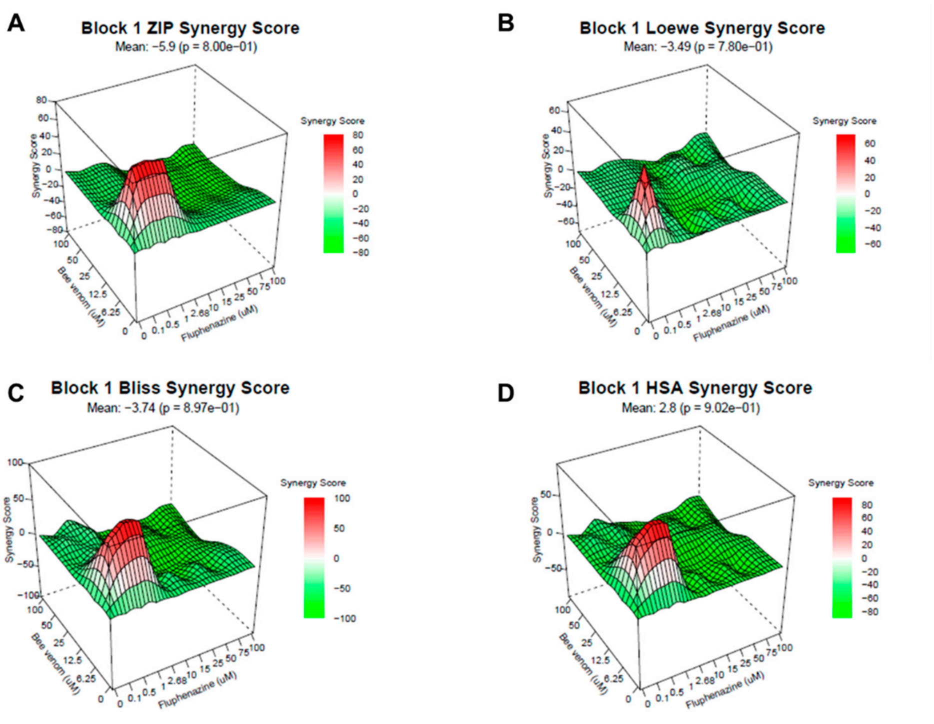

Figure S7. Synergy plots of combined treatments of fluphenazine combined with honeybee venom on MCF-7 breast cancer cells calculated with (A) ZIP; (B) Loewe; (C) Bliss and (D) HAS reference models. These data were obtained using SynergyFinder software. Synergy scores < -10, from -10 to 10 and > 10 indicates antagonism, additivity and synergism, respectively.

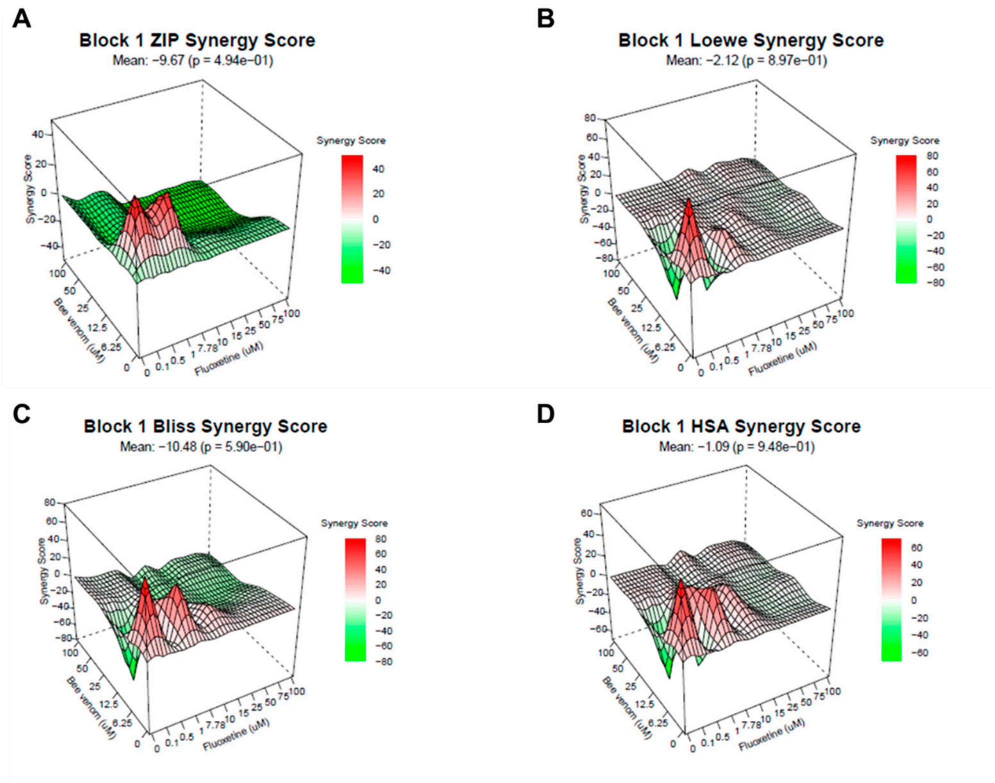

Figure S8. Synergy plots of combined treatments of fluoxetine combined with honeybee venom on MCF-7 breast cancer cells calculated with (A) ZIP; (B) Loewe; (C) Bliss and (D) HAS reference models. These data were obtained using SynergyFinder software. Synergy scores < -10, from -10 to 10 and > 10 indicates antagonism, additivity and synergism, respectively.

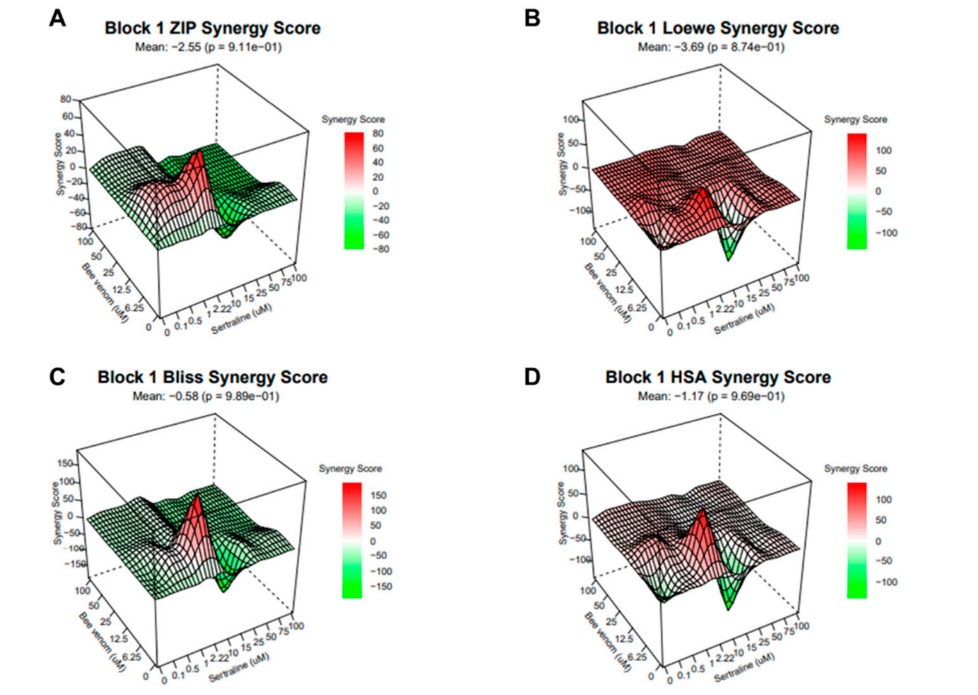

Figure S9. Synergy plots of combined treatments of sertraline combined with honeybee venom on MCF-7 breast cancer cells calculated with (A) ZIP; (B) Loewe; (C) Bliss and (D) HAS reference models. These data were obtained using SynergyFinder software. Synergy scores < -10, from -10 to 10 and > 10 indicates antagonism, additivity and synergism, respectively.

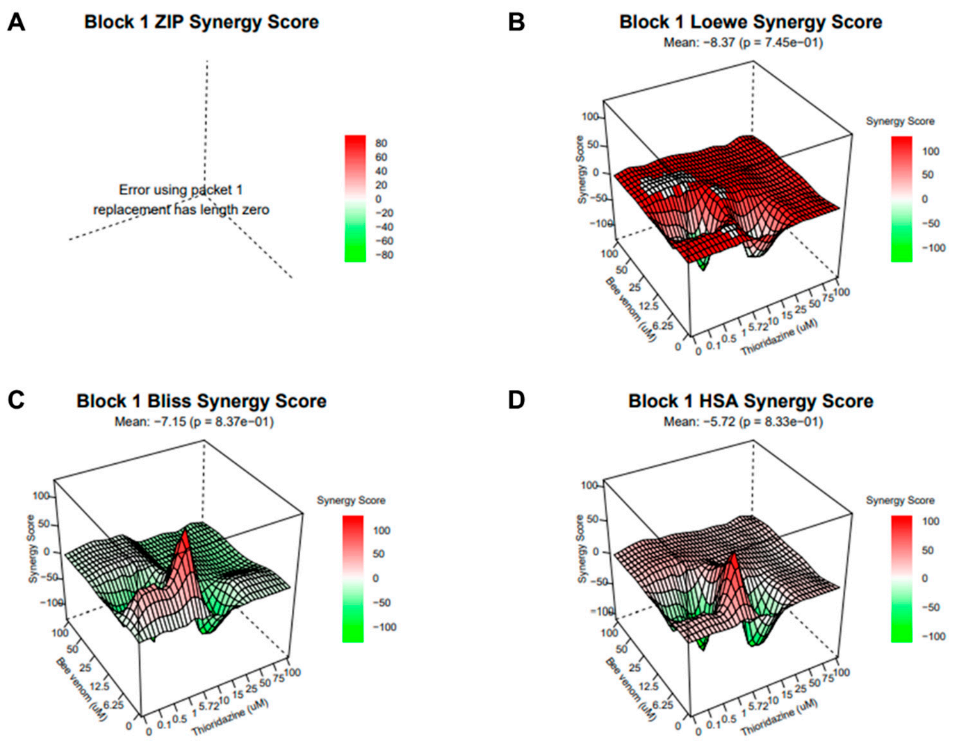

Figure S10. Synergy plots of combined treatments of thioridazine combined with honeybee venom on MCF-7 breast cancer cells calculated with (A) ZIP; (B) Loewe; (C) Bliss and (D) HAS reference models. These data were obtained using SynergyFinder software. Synergy scores < -10, from -10 to 10 and > 10 indicates antagonism, additivity and synergism, respectively.

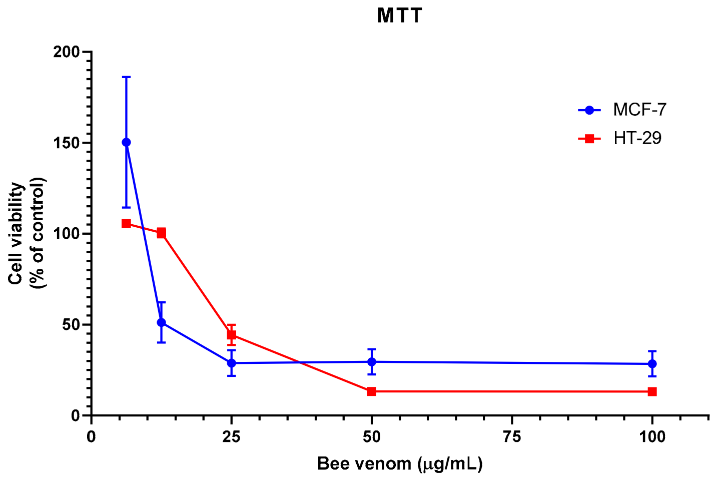

Figure S11. Comparison of the cytotoxic effect of increasing concentrations of honeybee venom in MCF-7 and HT-29 cancer cells. Cells were cultured in 96-well plates and treated with increasing concentrations (6.25-100 μg/mL) of honeybee venom for 48 h. Each point represents the mean ± SEM relative to the control cells (0.1% DMSO) of three independent experiences.

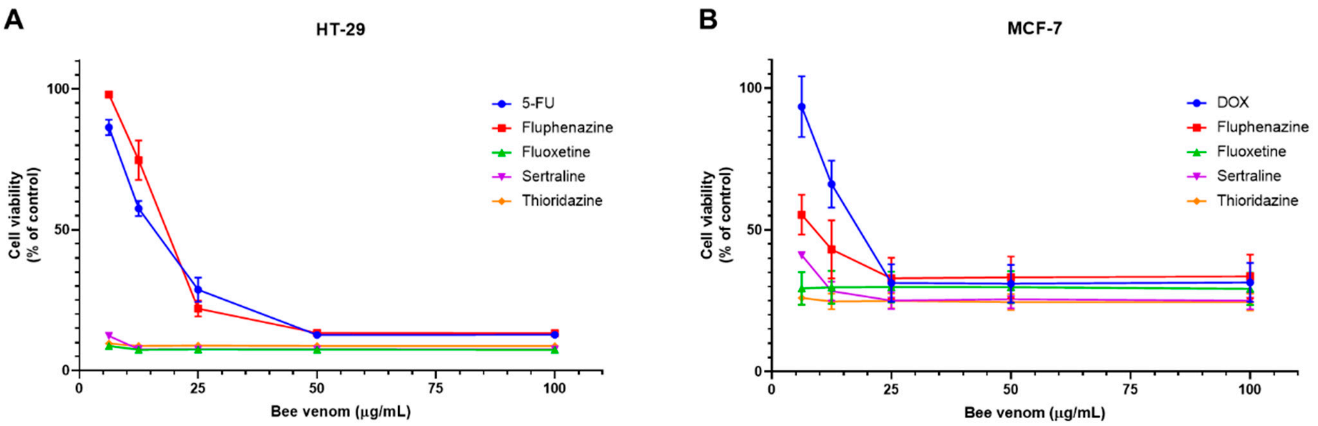

Figure S12. Comparison of the cytotoxic effect of honeybee venom combined with antineoplastic drugs and CNS drugs in (A) HT-29 colon and (B) MCF-7 breast cancer cells. 5-FU and DOX were used as reference drugs for HT-29 and MCF-7 cancer cells, respectively. Cell viabilities were determined after the final treatment with MTT assay. Each point represents the mean ± SEM relative to the control cells (0.1% DMSO) of three independent experiences.

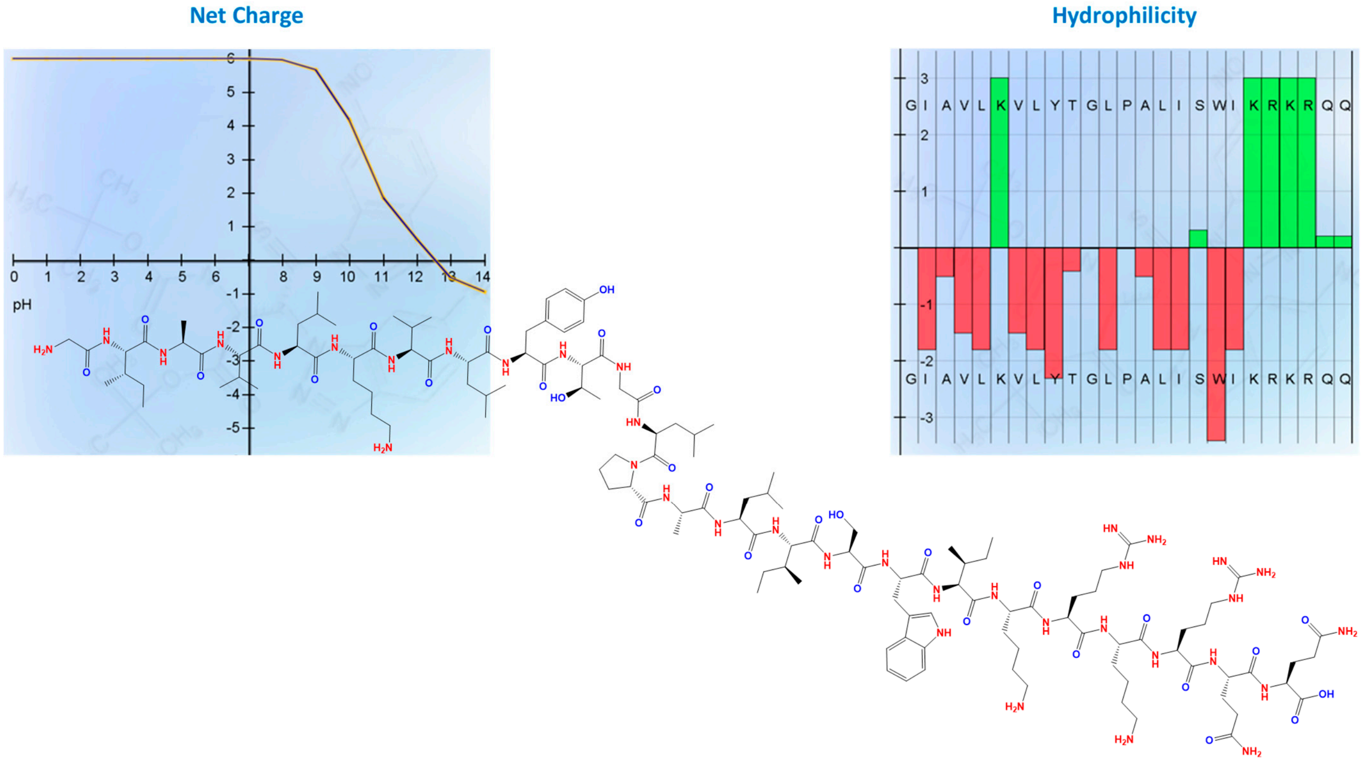

Figure S13. Structure of peptide MEL, melittin, with the sequence in 3-letter code of Gly-Ile-Ala-Val-Leu-Lys-Val-Leu-Tyr-Thr-Gly-Leu-Pro-Ala-Leu-Ile-Ser-Trp-Ile-Lys-Arg-Lys-Arg-Gln-Gln, and N-terminus with H- and C-terminus with –NH2. At the top of the image, the peptide properties are calculated with sequence-based functions of Peptide Calculator from BACHEM (https://www.bachem.com/knowledge-center/peptide-calculator/) for a net charge of pH = 7 and hydrophilicity with the Eisenberg scale. The molecular weight of MEL is 2851.52 g/mol, isoelectric point is 12.5, net charge at pH 7.0 is 6.0, average hydrophilicity is −0.3 and the ratio of hydrophilicity residues/total number of residues is 32%.
